# A self-assessment maturity matrix to support large-scale change using collaborative networks in the New Zealand health system

**DOI:** 10.1186/s12913-024-11284-6

**Published:** 2024-07-24

**Authors:** Kanchan M. Sharma, Peter B. Jones, Jacqueline Cumming, Lesley Middleton

**Affiliations:** 1 Te Tai Ōhanga The Treasury, 1 The Terrace, Wellington, 6011 New Zealand; 2 Department of Medicine, Faculty of Medical and Health Sciences, University of Auckland, 34 Princes Street, Auckland CBD, Auckland, 1010 New Zealand; 3 Health Services Research Centre, Faculty of Health, Victoria University of Wellington, Kelburn Parade, Kelburn, Wellington, 6012 New Zealand; 4 Faculty of Health, Victoria University of Wellington, Kelburn Parade, Kelburn, Wellington, 6012 New Zealand

**Keywords:** Large-scale change, Health care reform, Maturity matrix, Continuous improvement, Integrated care, Collaborative networks

## Abstract

**Background:**

A maturity matrix can be a useful tool for organisations implementing large-system transformation (LST) initiatives in complex systems. Insights from implementation of a local LST initiative using collaborative networks, known as Alliances, highlighted a tool was needed to help health system leaders prompt discussions on how and where to focus their change efforts. In the New Zealand (NZ) health system, Alliances were introduced to integrate the planning and delivery of health care between primary and hospital care.

**Methods:**

The aim of this research was to use insights from Alliance members to develop a learning tool that collaborative networks could use to assess and improve their readiness for change. We constructed a maturity matrix using the knowledge of senior NZ health system leaders, in a workshop setting. The maturity matrix was empirically tested and refined with three Alliances and with feedback from the NZ Ministry of Health Māori Health Strategy and Policy team.

**Results:**

The maturity matrix described the 10 key elements that had been found to support successful implementation of LST initiatives in the NZ health system, along with success indicators and different stages of maturity from beginning to excellence. Testing of the maturity matrix with three Alliances suggested that it functioned as a learning tool and stimulated collective thinking and reflection. The Māori Health Strategy and Policy team commented on the importance of such a tool to increase health system leaders’ responsiveness to improving Māori health outcomes. Comparisons with similar international matrices revealed common elements with ours. A strength of our maturity matrix is that it is specific to the NZ context and is the first practical tool to implement large-scale change in the health system that incorporates principles of the Government’s treaty with Māori, the indigenous people of NZ.

**Conclusions:**

Through a regular self-assessment process, use of the maturity matrix may create feedback loops to support deliberate learning and knowledge sharing for senior health system leaders and collaborative networks. The maturity matrix fills an important gap in the NZ health system and contributes to implementation science literature internationally.

**Other:**

This study was approved by the Victoria University of Wellington Human Ethics Committee (Ethics Approval Number 27,356). The research was supported by the Victoria University of Wellington research grant (222,809) and from the University of Auckland Department of Medicine research fund (H10779).

**Supplementary Information:**

The online version contains supplementary material available at 10.1186/s12913-024-11284-6.

## Background

Research from the United Kingdom and Europe shows an emerging use of maturity matrices in health care settings as deliberate learning tools for organisations dealing with complex changes. These include uses in primary care and hospital settings to stimulate quality improvement and to improve communication and co-operation among teams; assess readiness for change; identify areas of improvement; share experiences; help organisations understand the local conditions that enable successful delivery of integrated care; and evaluate the delivery of integrated care [1–11]. Other uses of maturity matrices have included measuring performance or evaluating the success of interventions in specific health conditions or programmes, such as, evaluating the role of Cardiac Genetic Nurses in inherited cardiac conditions [12]; measuring success of health policies in local government [13]; and defining stages of development and maturity of medicine programmes in Canadian hospitals [14]. Evaluations on the use of maturity matrices confirm that a self-assessment matrix can be a useful tool to implement and sustain large-system transformation (LST) initiatives, as it helps health system agents recognise their strengths and weaknesses and identify areas of improvement needed for system transformation [1, 3, 4, 9, 11, 15].

In this article we report on the development of a maturity matrix: a learning tool specifically designed for trust-based collaborative networks, such as Alliances, to improve communication and co-operation among health system leaders, assess readiness for change and identify areas for improvement to successfully implement LST initiatives in health systems. The purpose of this maturity matrix is to prompt discussions among senior health system leaders on where best to focus attention along an improvement pathway when implementing complex interventions in a complex adaptive system. By LST initiatives, we mean ‘interventions aimed at co-ordinated, system wide change affecting multiple organisations and care providers, with the goal of significant improvements in the efficiency of health care delivery, the quality of patient care, and population-level patient outcomes’ (p 422) [16].

In 2015, the New Zealand (NZ) Ministry of Health (Manatū Hauora) implemented the System Level Measures (SLM) programme to enhance a collaborative way of working beyond organisational and professional boundaries, as well as to address health inequities and encourage continuous learning and quality improvement [17]. The SLM programme was designed by Manatū Hauora with health system leaders from primary and secondary care. Alliances were collaborative networks that were responsible for leading the implementation of the programme in their districts. The implementation included development of an annual improvement plan using a collaborative approach underpinned by robust improvement science, and monitoring and reporting progress against successive plans [17].

A maturity matrix was constructed as part of a doctoral thesis that investigated the journey of the SLM programme, to better understand how the different elements coalesced to drive improvement in a complex adaptive system [18, 19]. The research identified a set of 10 key elements needed in the NZ health system to increase the chances of success with implementation of LST initiatives. These are (i) an alliancing way of working; (ii) a commitment to te Tiriti o Waitangi^1^; (iii) an understanding of equity; (iv) clinical leadership and involvement; (v) involved people, whānau (family and extended family), and community; (vi) intelligent commissioning; (vii) continuous improvement; (viii) integrated health information; (ix) analytic capability; and (x) dedicated resources and time [18, 19].

The preparedness of teams to engage with a maturity matrix reinforces the Alliance teams’ interest in continuous improvement and accords with the value of actions that support a learning culture. Work by others tracking the conditions that support large-scale transformation highlights four system enablers that have overlaps with our 10 key elements: (i) build an authorizing environment; (ii) provide relevant, authentic, timely, and meaningful data; (iii) designate and distribute leadership and decision making; and (iv) support the emergence of a learning culture [20].

## New Zealand Alliances

At the time of this research, the NZ health system had 20 geographically based District Health Boards (DHBs) that delivered publicly funded hospital and specialist services and purchased primary care services from Primary Health Organisations (PHOs). DHBs funded PHOs (not-for-profit meso-layer organisations) to provide comprehensive primary care services through their member general practices. Citizens choose the general practice they enrol with and general practices choose which PHO to become a member of [21]. Manatū Hauora had overall leadership of the health and disability system. A simplified visual description of the NZ health system at the time of this research is shown in Supplementary Figure A.

An important context for the health and disability system relates to the rights and interests of Māori who are the indigenous population of NZ. The British Crown and Māori rangatira (chiefs) signed te Tiriti o Waitangi (the Treaty of Waitangi) for the populations to live together under a common set of laws and agreements [22]. Under te Tiriti o Waitangi (te Tiriti) principles, crown agents have responsibility to work together with iwi (Māori tribe), hapū (sub-tribe), whānau (family or extended family) and Māori to plan, develop, and deliver health and disability services. The purpose is to ensure Māori receive equitable health care and have equitable health outcomes as pākehā (non-Māori) while protecting Māori cultural concepts, values and practices [22, 23].

Since 2013, Manatū Hauora contractually required all DHBs and PHOs in each district to form Alliances to deliver integrated patient-centred health care [24, 25]. Manatū Hauora published an Alliance Charter, which outlined the rules of engagement that Alliance members pledged to [26]. It also outlined members’ commitment to act in good faith to develop an Alliance plan for their district and decide how to fund and deliver their agreed plan [26]. Members committed to actively engage in good faith, and honour confidentiality, shared responsibility, shared decision-making, and shared accountability, to enable open and transparent discussions [26].

Most DHBs had a single Alliance with PHOs in their district, the simplest being an Alliance between one DHB and one PHO. Some DHBs had multiple Alliances, typically for two reasons. The first reason was because a PHO provided primary care services in more than one DHB district resulting in one Alliance that involved the local DHB, the PHO and other local partners in a district and another Alliance with the PHO and all DHBs the PHO provided primary care services in. The second reason was where more than one PHO provided primary care services in a DHB district and, usually owing to their poor relationship and/or history of working together, the PHOs did not agree to forming a single Alliance. The DHB then formed separate Alliances with each of their PHOs.

Each Alliance was governed by an Alliance Leadership Team (ALT). ALT members were appointed by agreement between member DHBs and PHOs and were made up of senior operational and clinical leaders. Some ALTs included local iwi (Māori tribe), community representatives, and other health service providers such as ambulance services, pharmacy, and Māori and Pacific health providers. The most common type of ALT consisted of the DHB Chief Executive and/or planning and funding manager, the PHO Chief Executive, and DHB and PHO clinical leaders.

NZ Alliances were not legal entities and therefore could not commission (fund and contract for) services, nor did they have their own budgets for spending on health care. Instead, Alliances had access to a flexible funding pool, a portion of PHO funding set aside to provide management services, health promotion activities, services to improve access, services to manage chronic care in the community, and support rural health providers, and they could use this funding pool to support new initiatives [25]. ALTs agreed on a shared vision and goals for their local health system and agreed a work programme with their Alliance partners. DHBs were encouraged to contribute additional funding to the flexible funding pool to support the Alliance work programme [25].

NZ Alliances adapted over time, depending on local relationships, interactions, behaviours, and their history of working together; they therefore varied across the country in form, function, and maturity. For example, in response to the SLM programme, the three DHBs and the seven PHOs providing health services across the largest city, Auckland, formed a single Alliance.

Two authors (KMS and PBJ) led the development and implementation of the SLM programme from Manatū Hauora and gained first-hand insights and knowledge on the inner workings of NZ Alliances. They identified three clusters of Alliances as a result of their day-to-day interactions over a period of five years and through assessing the improvement plans and monitoring Alliances’ progress with implementation of the SLM programme.

First, there were high-functioning Alliances. These were seen to have an agreed shared vision and common goals for their local system, were clinically led, had an independent chair, and placed people and their communities at the centre of their decision-making. The high-functioning Alliances established Service Level Alliance Teams (SLATs) or other informal working groups, such as consumer councils and clinical leadership forums, as necessary to deliver on the Alliances’ work programme. SLATs were workstreams within the Alliance structure (e.g., child health SLAT, youth health SLAT, rural SLAT) and reported to the Alliance. Each SLAT was made up of a diverse group of people relevant to the workstream that included clinicians, managers, analysts, service users, and Māori and Pacific representatives. SLATs used improvement science methods to identify problems and co-design solutions to improve health service delivery. ALTs considered recommendations from SLATs and then made recommendations to the DHB executive team on activities and services to meet the Alliance vision and goals. Upon agreement, DHBs and PHOs commissioned change through their contractual processes to give effect to the Alliance priorities [25]. ALTs monitored outcomes of Alliance activities and fed the information back to their member organisations. They refreshed their work programme and membership at least annually.

The second cluster of Alliances existed simply to meet the contractual requirement for there to be an Alliance, and which allowed partner PHOs and their contracted providers access to the flexible funding pool. These Alliances were constrained by their capability to lead change and improvement initiatives and lacked insight as to their strengths and weaknesses. From our observations they did not know what a high-functioning Alliance looked like, how they compared with other Alliances, or what they needed to do to become high-functioning to implement and sustain LST initiatives.

The third cluster of Alliances were dysfunctional, either owing to a lack of leadership from the DHB and/or the PHO or a lack of understanding about the alliancing concept. These Alliances were seen to have had a poor history of working together and low-trust relationships between senior managers of DHBs and PHOs, and between senior managers and clinicians in DHBs and PHOs. They were further hampered by a lack of capability for improvement and therefore lacked awareness of their inability to be functional.

To help the second and third cluster of Alliances, we identified that a maturity matrix had the potential to focus attention on the features that will put the lesser performing Alliances on a developmental pathway to become high functioning. We expected a maturity matrix would enable Alliances to assess their readiness for change, measure improvement progress over time, and to identify their development areas. We aimed to:

- Search the literature for existing matrices that could be adopted for the NZ context.
- Use the insights from Alliance members to refine and develop a NZ specific maturity matrix.

## Methods

This research was conducted between November 2018 and December 2019 and included four phases.

### Phase 1 – Literature search

The published and grey literature was searched using keywords in the OVID and PUBMED databases using the keywords: maturity audit or checklist or matrix or framework or stages or self-assess or tool or models, stages of organisational maturity, and quality assurance or indicator. The search was limited to English language from 1946 to 2018. Grey literature was searched using Google and visiting known quality improvement websites in the United States, United Kingdom, Canada, Australia, and NZ. These included websites such as the King’s Fund, Nuffield Trust, Institute of Healthcare Improvement, and the Health Quality & Safety Commission. The search was further refined using keywords self-assessment and performance and was limited from 2008 to 2018. A total of 22 articles were identified from which 12 were deemed relevant and examined further.

Three maturity matrices were considered for use in the NZ health system: (i) a self-assessment maturity matrix used in Danish general practices to stimulate quality improvement, which was later refined and used in general practices across the UK and the Europe [1, 2]; (ii) the Development for Integrated Care (DMIC), a web-based self- evaluation tool used in Netherlands [9]; and (iii) the Scaling Integrated Care in Context (SCIROCCO) maturity matrix, an online self-assessment tool used across health and social care systems in the European Union [10]. Nine other matrices were studied for their conceptual frameworks for defining stages of maturity and measuring progress along the maturity scale that could be adapted for NZ [5, 7, 8, 12–14, 27, 28].

None of the 12 overseas maturity matrices studied were considered suitable to be adopted for use in the NZ health system. Most were limited in their scope to one service or setting, and while the SCIROCCO and DMIC maturity matrices had a multi-disciplinary team focus and contained useful domains, they did not feature rights of indigenous people nor considered an indigenous treaty or partnership such as te Tiriti o Waitangi.

Of the 12 maturity matrices examined, the conceptual framework and the development process to construct a maturity matrix for the NZ health system was adapted from Kirk, Simpson, et al. [12]. This study provided a simple and practical framework to construct a maturity matrix that described the outcome sought, key indicators to measure the outcome, and description of maturity along the scale of beginning, emerging, established, and excellence. We believed the consensus approach used by Kirk, Simpson, et al. [12] was a useful and pragmatic way to elicit information from research participants with limited availability who would also be users of the matrix.

### Phase 2 – Workshop

Senior clinical and operational leaders from the NZ health system constructed the maturity matrix in a workshop setting (*n* = 10). Participants involved were those with significant experience in the design and implementation of LST initiatives and those charged with making major strategic decisions about resourcing these initiatives in their organisations. These included DHB planning and funding managers, PHO Chief Executives, hospital and primary care clinical leaders, senior managers from Manatū Hauora and the Health Quality and Safety Commission, and Māori and Pacific community leaders who held senior roles in the health system.

The workshop was facilitated by one of the authors (PBJ) who was the SLM Programme clinical lead and had the relevant skills, subject matter expertise and credibility to elicit information from participants. Table 1 shows the framework, adapted from Kirk, Simpson, et al. [12], that was used to construct the maturity matrix. Participants worked in groups to identify the outcomes, success indicators and the maturity scale for all 10 elements: (i) an alliancing way of working; (ii) a commitment to te Tiriti o Waitangi; (iii) an understanding of equity; (iv) clinical leadership and involvement; (v) involved people, whānau (family and extended family), and community; (vi) intelligent commissioning; (vii) continuous improvement; (viii) integrated health information; (ix) analytic capability; and (x) dedicated resources and time. [18, 19]. An iterative approach was used to reach consensus among participants.

**Table 1:** Maturity matrix outline adapted from Kirk, Simpson, et al. [12]

Key element 1					
Outcome descriptor	**Indicators (what will show this? )**	**Maturity scale**			
		Beginning = 0	Emerging = 1	Established = 2	Excellence = 3
What does established element demonstrate?	Whole-of-system indicator	Nothing in place	Something in place	This is what good looks like	Outstanding e.g., health and social integration
	Equity indicator	Nothing in place	Something in place	This is what good looks like	Outstanding e.g., health and social integration
	Te Tiriti o Waitangi indicator	Nothing in place	Something in place	This is what good looks like	Outstanding e.g., health and social integration

The first version of the maturity matrix constructed at the workshop was shared with workshop participants following the workshop for their feedback. The second version of the maturity matrix that incorporated post-workshop feedback from participants was used for field testing with the ALTs.

### Phase 3 – Field testing

The aim of this phase was to empirically test the maturity matrix with three ALTs that represented the three clusters. The test aimed to determine the extent to which the maturity matrix functioned as a learning tool, helped Alliances see where they were on the improvement journey and identify areas of improvement, acted as a resource for collective thinking and reflection, and if it was easy and practical to use. ALTs were purposefully sampled based on the size of the population the DHB was serving, the number of PHOs providing services in the district, the membership of the ALT, and the knowledge and insights on the maturity and functionality of ALTs from the two authors (KMS and PBJ) involved in the development and implementation of the SLM programme. ALTs 1 and 3 had a broad membership that included senior DHB and PHO managers and clinicians, consumer advocates, and representatives from community health services such as pharmacy, Māori and Pacific providers and district nursing. ALT 1 had one PHO providing primary care services in the district while ALT 3 had multiple PHOs in their Alliance. ALT 2 had limited membership with only DHB and PHO managers and clinicians. The order of input was determined by the ALTs’ availability to meet for the testing.

The testing process was facilitated by one of the co-authors (PBJ) for ALTs 1 and 3. PBJ was unavailable to facilitate testing for ALT 2, so a workshop participant facilitated this session. KS was present at all three ALT testing sessions as an observer. Written consent was provided by all participants. Group discussions were used to collate feedback from participants on the content of the maturity matrix. Ideas for improvement from ALT 1 was tested with ALT 2, and ideas from ALTs 1 and 2 were tested with ALT 3 to discuss different perceptions, consolidate ideas, and to refine the maturity matrix. Some changes were made to the matrix as it progressed through the testing with three ALTs to improve efficacy. The final version of the matrix was shared with all three ALTs. This is fully discussed in the results section.

### Phase 4 – Input from Manatū Hauora Māori Health Strategy and Policy (MHSP) team

**Fig. 1:**
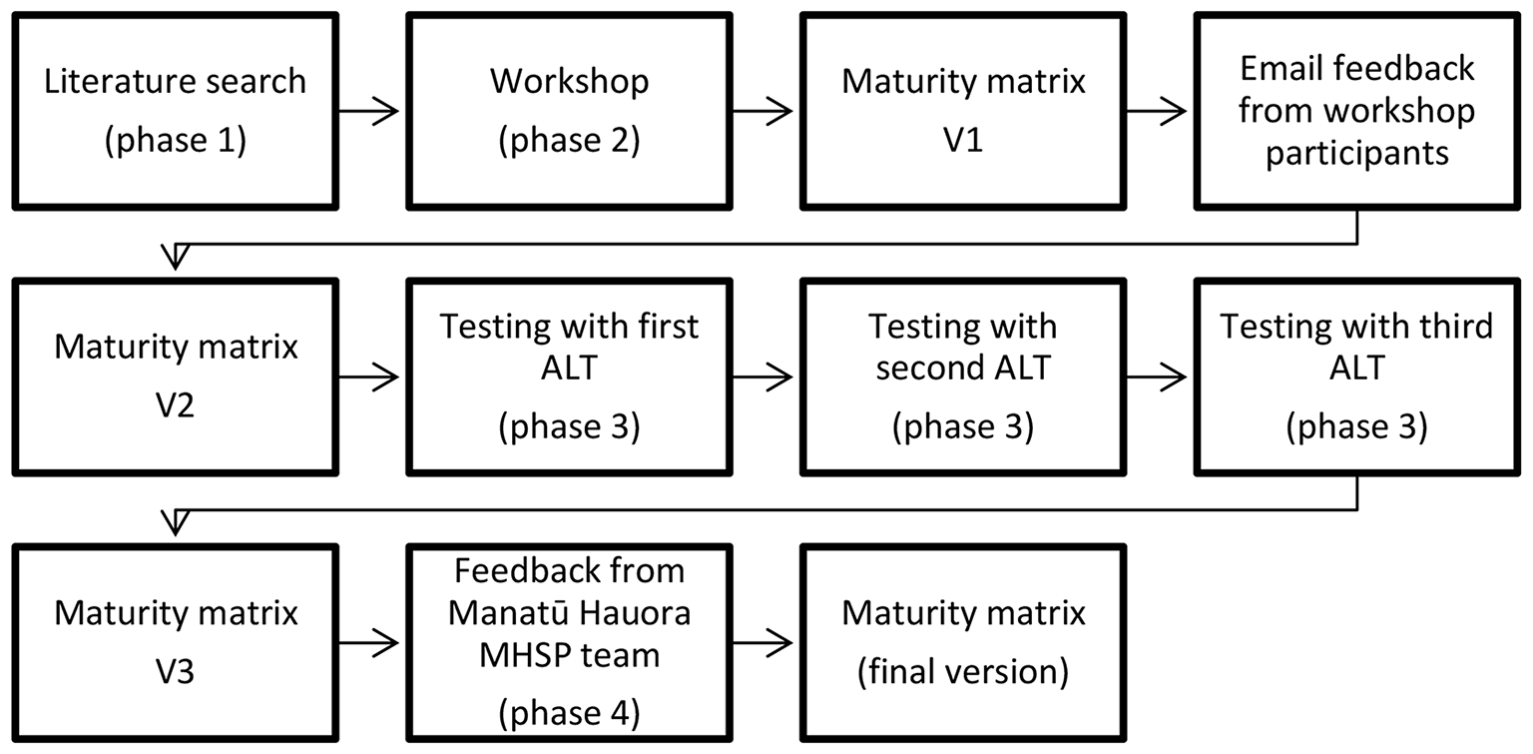
Iterative process for developing and refining the maturity matrix

Following testing with the three ALTs, the maturity matrix was revised and shared with Manatū Hauora MHSP team to seek their feedback on the matrix to ensure that te Tiriti principles were accurately reflected across the maturity scale for all the key elements, with particular attention to two elements: commitment to te Tiriti o Waitangi; and understanding of equity. All feedback was incorporated, and the revised maturity matrix was shared with the MHSP team manager to ensure its accuracy, which was confirmed. Figure 1 summarises how the maturity matrix was developed, tested, and refined over the four phases.

## Results

Results were drawn from phases three and four of the testing (Fig. 1). Testing of the maturity matrix with the ALTs was an important part of the research process. It allowed us to check if the matrix covered all the key elements that Alliances believed were important to support them with implementation of LST initiatives; if the level of detail enabled the ALTs to assess their capability; and if the self-assessment process stimulated collective thinking and reflection on contextual factors that enable or constrain Alliances’ success with change efforts.

The version tested with the ALTs (V2) had scores for each maturity stage, i.e., beginning = 0, emerging = 1, established = 2, and excellence = 3. The scores, along with other structural components of the matrix (the conceptual framework, 10 elements, and maturity scales), remained unchanged throughout phase 3. However, there were changes made following each ALT testing to improve the layout (e.g., adding a row to add up scores for each element), descriptive details of elements and their outcomes, and the tone of the matrix to be empowering and inclusive. The iterative approach allowed us to improve the efficacy of the matrix without making substantive changes to the design and core content.

All ALTs rigorously debated the scores, for example, whether they were 1 or 1.5 and some participants found it challenging to look beyond their organisation when scoring. ALT 3 recommended removing the scores to take the focus away from getting the ‘right’ score and focussing on improvement instead. The ALT felt the scores were meaningless and removing them shifted the conversation to factors important for transformation such as, leadership, trust, and relationships.

*As a suggestion for the tool*,* remove the numbers. Because you’re not a two or three*,* the power is in the words and the discourse and what you’ll end up with is people ending up with is people averaging across three of the sections and say*,* we’re a 2.33 and that is absolutely meaningless. That’s what happens if you put numbers on these sorts of scales that people do that. So*,* if you take the numbers off then people have to use words and the words mean something.* (ALT 3 participant).

The testing process with ALTs was influenced by local contextual factors. For example, for ALT 1, which had high-trust relationships and a positive history of working together, the focus of testing was on their readiness for change and to identify areas of improvement for the Alliance. The ALT was motivated to use the matrix regularly for continuous improvement. In ALT 2, which did not have a history of working together constructively and where trust was low between the DHB and service providers, the focus was on getting the right score, debating the maturity scale, and whether a larger range of scores was needed to enable Alliances to accurately score. Further, this Alliance placed an emphasis on performance of individual providers or Alliance member organisations rather than a collectively assessment of their Alliance. For example, DHB and PHO members blaming each other for past failures of the Alliance’s efforts. We also observed a lack of trust between DHB/PHO management and clinical leaders, and Manatū Hauora (i.e., suspicion that Alliances’ results from the assessment would be used against them, or at least, for judgement of ALT performance). ALT 3 was initially reticent to use the matrix as they believed they were already high functioning and therefore did not need to self-assess against a tool. We observed a command-and-control leadership style of DHB members in this Alliance. However, once the facilitator started the testing process and encouraged participation from non-DHB members, the ALT completed the self-assessment process, identified areas of improvement and said that they found the tool useful to reflect and stimulate open and honest communication among the members.

The feedback from ALTs helped improved consistency of language and tone used in the matrix to strengthen the intent of maturity matrix as a learning tool that is empowering, inclusive of all health system agents and focuses on wellbeing outcomes (rather than just health outcomes).

The three ALTs agreed that the key elements provided adequate coverage of the areas considered important to the Alliance and that the maturity matrix stimulated collective thinking, reflection, and improvement, “…. *certainly*,* a useful exercise to go through and it makes you think and reflect on where you’ve got to and where you might go to next*” (ALT 3 participant). Table 2 summarises key suggestions from ALTs to improve the matrix.

**Table 2:** Key suggestions from ALTs to improve the maturity matrix

Theme	Key suggestions
Descriptive detail, tone, and audience of the matrix	ALT 1 suggested we provided clear definitions for each element in the matrix. ALT 2 suggested we added more narrative to outcome descriptors to enhance the improvement story. ALT 1 suggested changing the tone and language used in the matrix so it’s empowering, inclusive, and wellbeing focused. ALTs 2 and 3 suggested we clarified that the matrix was for the Alliance members to assess their Alliance (and not for the ALT to assess individual providers/partners of the Alliance).
Te Tiriti reference	ALT 1 suggested references to te Tiriti in the matrix was in Te reo Māori so it referred to the Māori version (and not the English version that has different interpretation for Māori and Pākehā).
Scores for each maturity stage	ALT 2 suggested we removed zero as a score and included a score range so Alliance can accurately score, i.e., 1–3 for beginning, 3–6 for emerging and so on. ALT 3 felt the scores were a distraction and that removal of scores would focus the discussion on improvement and away from getting the ‘right’ score.
Highlighting the importance of trust	ALT 2 felt high trust was the most important tenet for the ‘alliancing way of working’ element and should be identified in the description and as an outcome for this element and that it needs to be sustained at an excellence stage.
Continuous improvement	ALT 2 suggested we include an action plan at the end of the matrix for ALTs to identify, document and prioritise their next steps on key areas of development.
Fidelity of the matrix	ALT 3 suggested more rigorous testing of the matrix, including national benchmarking to ensure national consistency in the assessment process and sharing of results to see how they compare with their peers.

### Feedback from Manatū Hauora Māori health and strategy (MHSP) team

Manatū Hauora MHSP team reviewed version five of the maturity matrix (following testing with ALTs) from an equity and te Tiriti perspective and provided feedback. Overall, their feedback was positive and commended equity and te Tiriti being embedded in the maturity matrix, both as key elements and as indicators of maturity. They commented on the necessity of a maturity matrix such as this to increase the understanding and responsiveness of health system leaders and Alliances towards fulfilling their obligation to te Tiriti and improving Māori health outcomes. Table 3 summaries feedback from the team to strengthen descriptions in the matrix and to strengthen the alignment with Whakamaua, the Māori Health Action Plan.

The final version of the maturity matrix is supplied as Additional file 2 – Table A.

**Table 3:** Summary feedback from Manatū Hauora Māori Health and Strategy team

Key element	Feedback
Commitment to te Tiriti o Waitangi	That both Māori and iwi are acknowledged in the matrix because iwi is te Tiriti partner and not Māori. Not all Māori associate with an iwi. That Māori community and Māori/iwi-led service providers are distinguished in the matrix. Voting right for Māori should be reflected in the ‘established’ stage rather than the ‘excellence’ stage as it is what should be expected as a norm. The outcome descriptor should aim to address and eliminate institutional racism rather than just aiming to reduce, and historical contexts for institutional racism and injustices need to be understood at the ‘beginning’ stage.
Involved people, whānau and community	The maturity scale should show the complexity of iwi and Māori involvement in the design of solutions, e.g., from tokenistic consultation at the beginning stage to a co-design with decision-making rights over the final solution at the excellence stage.
Integrated health information	The maturity scale could be clarified to reflect Alliance having access to: basic data at national level (by age and gender) at the ‘beginning’ stage; broad ad hoc data disaggregated by ethnicity with recognition of Māori data sovereignty at the ‘emerging’ stage; a shift away from ad hoc data collection and analysis by ethnicity to routine, mandatory reporting and monitoring by ethnicity at the ‘established’ stage; and use of Integrated Data Infrastructure to connect health and social data at the ‘excellence’ stage.
Intelligent commissioning	The maturity scale could be clarified to reflect prioritisation of resources in proportion to Māori population need and risk so there is a tailored and targeted approach to actively protecting Māori health and wellbeing, including protecting Māori and iwi-led health providers.
Continuous improvement	The maturity scale could include establishment of Māori measures of health and wellbeing as defined by iwi and Māori.

## Discussion

Testing with ALTs revealed that the maturity matrix stimulated collective thinking and reflection for Alliances on key elements and conditions that increase chances of success with implementation of LST initiatives. The matrix acted as a compass for Alliances to see where they were along the maturity scale and identify areas of improvement. The self-assessment process could be used prospectively to gauge readiness for change, in real time when implementing change, and retrospectively to understand failures or partial successes of change efforts, a finding supported by evaluation of maturity matrices used in other settings [1, 3, 4, 9, 11, 15]. A continuous use of the self-reflection process, along with key actions to improve, should build capability of health system leaders and networks to implement and sustain LST initiatives [8, 12–15, 27, 28].

Having a non-judgemental facilitator who was familiar with the maturity matrix and had creditability with ALTs proved to be an important enabler of the testing process, a finding supported by evidence in literature [4]. The facilitator was able to assist with interpretation of the maturity matrix and move teams along if they got stuck on one element or indicator. The facilitator’s credibility was important as this meant that ALT members knew the facilitator, their experience in the health system and the history of their way of working. This knowledge and experience created trust with ALTs and enabled them to assess their strengths and weaknesses sincerely and not worry about presenting their better side or being judged on their discussions or results of their assessment.

The elements, maturity levels, and the self-assessment approach of this maturity matrix can be compared to the two international matrices designed to help organisations understand the local conditions that enable successful delivery of integrated care (the DMIC tool [9] and the SCIROCCO project [10]).

The DMIC tool focuses on delivery of integrated care for a condition (such as stroke or diabetes services) at an organisational level. It is comprehensive with a total of 89 elements grouped in nine clusters. This tool is different to our maturity matrix as it is designed for those involved in the delivery of services (co-ordinators, managers and professionals) to develop, evaluate and improve the delivery of integrated care. Ours is designed to implement LST initiatives that are broad and widespread across geographical and professional boundaries, seek paradigm shifts in mindsets and relies on building and sustaining high trust relationships among senior system leaders who oversee design, funding and delivery of health services.

The SCIROCCO is a project co-funded by the Health Programme of the European Union. It is a self-assessment tool that enables those working in the health and social care system across the European Union to assess their readiness to deliver integrated care. The tool aims to help European regions to understand their strengths, weaknesses and potential areas of improvement, adopt and scale up integrated care solutions, facilitate multi-stakeholder discussions on progress and delivery of integrated care and facilitate coaching to help regions and organisations understand the local conditions that enable successful delivery of integrated care. One of the important components of this model is that regions share their experience and assessments with others through a web-based platform. This facilitates sharing of knowledge between the regions.

Elements common across these two matrices and ours include involvement of citizens, a continuous improvement approach, a population health approach to address health inequities, a collaborative way of working, integrated data and analytical capability, integration of health and social care services, and dedicated resources in the form of funding, time, and change management teams.

All three matrices aim to facilitate discussions among multi-disciplinary teams to build a shared understanding of their readiness to implement LST initiatives in the health system and to identify areas of improvement.

We considered adopting the SCIROCCO matrix for NZ, however health system leaders at the workshop felt that while the SCIROCCO domains were useful and related to the key elements they had identified, the assessment scale of the model contained generic statements that did not have sufficient detail to enable self-assessment for Alliances. This matrix did not encompass the NZ context, especially te Tiriti.

A strength of our maturity matrix is that it is specific to the NZ context and is underpinned by the principles of te Tiriti and equity in a multifaceted way by identifying them as separate elements and as indicators for each element. There are frameworks that outline how NZ government agencies should engage with Māori [29, 30], however, this maturity matrix is the first that provides a practical tool incorporating te Tiriti principles for collaborative networks to use to implement and sustain LST initiatives in the health system.

The 2022 reform of the NZ health system replaced Alliances with localities. Localities are local networks comprising of local health service providers, social sector agencies, non-government organisations, iwi, local authorities, and consumers and communities [31]. Te Whatu Ora - Health NZ has a legislative responsibility to ensure all of NZ is covered by a locality, that there is a plan outlining priority outcomes and services for the locality, and that Te Aka Whai Ora – Māori Health Authority and Iwi Māori Partnership Boards are involved in the development, implementation and review of the locality plan [32]. Localities are responsible for working together to meet local health needs and wellbeing outcomes for their population [32]. Localities are not legal entities, do not have a budget, and cannot commission services. Like Alliances, localities are mandated from the centre and will be guided by national policies and operating rules. Te Whatu Ora is responsible for commissioning services to deliver on the locality plans.

However, the new Government elected in October 2023 disestablished Te Aka Whai Ora with the intent of moving decisions closer to the community, home and the hapū [33]. It is unclear how functions of Te Aka Whai Ora will be incorporated across Te Whatu Ora and Manatū Hauora and what will become of localities.

Nonetheless, regardless of how the health systems are structured, collaboration between primary and secondary care, and other providers to improve delivery of health services, outcomes and equity remain important. In a recent NZ study, some DHB and PHO leaders said that Manatū Hauora’s mandate for them to form Alliances resulted in successful implementation of integrated work programmes that shifted siloed thinking [34].

While those with a positive history of working together or a willingness to share power and resources to improve outcomes for their population will continue to see the benefits of collaborative networks, there are those that will need a push in a form of mandate (push) or incentive (pull). The form and name of these collaborative networks are not important. It is the depth of processes, strong relationships, a high-level of trust, and the ability to work collectively towards a shared vision that add value to and success of these networks. These behaviours cannot be driven from the centre and will require iterative practice cycles that include an ability to collectively self-reflect, assess strengths and weaknesses, and learn. The iterative practice cycles create feedback loops and facilitate conscious and deliberate learning that refines and distributes knowledge gained by experience in a practical way. Collaborative networks, in whichever form they exist, will benefit from tools like this maturity matrix to foster deliberate learning and knowledge sharing to help them perform as cohesive high-functioning networks and to develop and deliver on their plans.

A limitation of this maturity matrix is that it was constructed with a small group of people and tested with three ALTs. More work is required to test, improve, and increase its fidelity, accessibility, and adoption. However, development of this maturity matrix makes an important contribution to implementation science literature in and beyond the NZ health system.

## Conclusions

This research broke new ground for NZ health system with the creation of a tool in the form of a self-assessment maturity matrix using the knowledge of senior system leaders to increase chances of success with implementation of LST initiatives. The maturity matrix provides an important tool for collaborative networks to support deliberate learning and knowledge sharing in a practical way. LST initiatives are not short on excellent conceptual and theoretical work on why improvement matters and how networked governance can support change. The contribution made by this work is to profile the value of supporting leaders with tools to manage the hard task of reaching and sustaining a state of maturity across all the elements needed to embed change.

### List of abbreviations and te reo Māori translations

**Table:** 

Abbreviations	Te reo Māori translations
ALT - Alliance Leadership Team DHBs – District Health Boards LST initiatives – Large-system transformation initiatives New Zealand – NZ PHOs – Primary Health Organisations SLAT – Service Level Alliance Team SLM programme – System Level Measures programme	Hapū – Māori sub-tribe Iwi – Māori tribe Manatū Hauora – Ministry of Health Māori – indigenous people of NZ Pae ora – Healthy futures Pākehā – New Zealanders of European descent or non-Māori Tangata whenua – the indigenous Māori people of a particular area of New Zealand or of the country as a whole Te Aka Whai Ora – Māori Health Authority Te reo Māori – Eastern Polynesian language spoken by the Māori people Te Tiriti o Waitangi – The Treaty of Waitangi Te Whatu Ora – Health NZ Whakamaua – Māori Health Action Plan Whānau – family or extended family Whānau ora – healthy families

### Electronic supplementary material

Below is the link to the electronic supplementary material.

Supplementary Material 1

Supplementary Material 2

## Data Availability

No datasets were generated or analysed during the current study.

## Footnotes

1 The Treaty of Waitangi was signed between the British Crown and Māori rangatira (chiefs of the indigenous population of NZ) for the populations to live together under a common set of laws and agreements.
