# Supplementary material for: A self-assessment maturity matrix to support large-scale change using collaborative networks in the New Zealand health system
Source: BMC Health Serv Res. 2024 Jul 24;24:838. doi: 10.1186/s12913-024-11284-6 (PMC11271159; doi:10.1186/s12913-024-11284-6)
Supplement: Supplementary file 2 — Supplementary Material 2 [file 12913_2024_11284_MOESM2_ESM.docx]

**Table A - Maturity matrix to support implementation of large-scale change in the New Zealand health system**

| Key element 1 - Alliancing way of working  Alliancing is a clinically, community and iwi-led forum that brings all agents in the health system together with the aim of transforming services with people and equity at the centre of their decision-making. Alliancing is a collaborative way of working, reliant on high-trust relationships and is supported by enablers critical to continuous improvement. Successful alliances serve the interest of the community while preserving the autonomy of multiple organisations and services present in a complex system. | | | | | |
| --- | --- | --- | --- | --- | --- |
| Outcome descriptor (what does the established element demonstrate?) | **Indicators (What will show this?)** | **Maturity scale – what we expect to see at each stage** | | | |
|  |  | **Beginning** | **Emerging** | **Established (this is what good looks like)** | **Excellence (outstanding eg health and social integration)** |
| System leaders demonstrate distributed leadership through a broad and inclusive alliance membership. There is high trust, shared vision, and sustained commitment from all alliance members | Alliance is structurally embedded in the system (accountable and visible) | Recognition and acknowledgement of the need to have a shared vision | Shared vision and goals,  roles, responsibilities, and accountability understood | Alliance is structurally embedded in the system with clear mandate, accountability, and dedicated resources. High trust exists among alliance members | High trust and shared vision are sustained with changes in membership and evolution |
|  | The manner in which all priority populations have input into decision making of the alliance | Little or no evidence of input  Tokenism or lip-service | Some populations influence decision-making | Multiple populations influence decision-making via different mechanisms  Evidence of innovation | Alliance reflects local community and includes social sector partners |
|  | Depth of commitment to partnership with Māori in all aspects of alliance activity | Māori representation (not partnership) in alliance | Key Māori/iwi leadership and partnership at the alliance within the context of te Tiriti o Waitangi | Te Tiriti o Waitangi principles embedded in the philosophy and operation of the alliance | Full commitment to multi-sectorial and -dimensional (holistic) view that enables Māori aspirations e.g., whānau ora approach |
| Alliance members are working together with a collaborative approach beyond their organisational and professional boundaries towards the shared vision | All members ‘live’ the alliance charter | Setting up an alliance charter | Some elements of the alliance charter are demonstrated or by some members | All members of alliance ‘live’ the alliance charter constantly and consistently | Alliance is achieving the shared vision while preserving the autonomy of individual providers |
|  | The degree to which organisations with a concern for priority populations are included in integrated programme (design, implement and evaluate) | Little if any involvement of appropriate organisations (only DHBs and PHOs involved) | Beginnings of integration within health alone including relevant organisations that serve priority population groups | Widespread integration with relevant health and disability NGOs with priority population interest  Beginnings of de-siloed funding | Collaboration within and beyond health to appropriate priority population groups including design, implementation, and evaluation of programmes  Mature de-siloed funding |
|  | Acknowledging iwi/Māori as the te Tiriti o Waitangi partner | Little or no engagement with Māori community and Māori and iwi-led health providers | Māori community and Māori and iwi-led health providers are consulted during the decision-making process of the alliance | Established Māori partners (iwi, hapū, providers etc) actively involved in the decision-making process of the alliance, including a voting right | Māori/iwi are leading the decision-making process in the alliance leadership teams, including Māori and iwi-led health and social care providers |
| Areas to work on: | | | | | |

| Key element 2 – Commitment to te Tiriti o Waitangi  Recognising the principles of te Tiriti o Waitangi through equity and active protection to achieve equitable health outcomes for Māori; culturally appropriate health care that recognises and supports Māori models of care, and working in partnership with Māori in the governance, design, delivery and monitoring of health services^[[1]](#footnote-1)^. | | | | | |
| --- | --- | --- | --- | --- | --- |
| Outcome descriptor (what does the established element demonstrate?) | **Indicators (What will show this?)** | **Maturity scale – what we expect to see at each stage** | | | |
|  |  | **Beginning** | **Emerging** | **Established (this is what good looks like)** | **Excellence (outstanding eg health and social integration)** |
| Alliance is working in partnership with Tangata Whenua in the governance, design, delivery, and monitoring of Māori health outcomes. Institutional racism is acknowledged and actions to eliminate are prioritised | Decision-making processes recognise, acknowledge, and correct for the impact and role of institutional racism | Understanding of historical contexts and injustices | Explicit acknowledgement of te Tiriti responsibilities and aware of alliance’s role to take proactive steps to address institutional racism | Alliance is taking actions to address institutional bias and improving Māori health outcomes in partnership with Tangata Whenua | Alliance work programme reflects an equitable system by design that includes principles of options and active protection |
|  | Degree to which te Tiriti is understood in the modern-day health system (ie biculturalism in a multi-culturalism context) | Understanding of root causes of inequities within the context of te Tiriti | Alliance is able to conduct basic analysis to understand inequities for Māori (ie by age and gender) | Alliance is using inter-sectional analysis to drive key actions to address inequities within the context of te Tiriti | Evidence of re-prioritisation of activities and resources for Māori within the context of te Tiriti, which leads to documented improved health outcomes for Māori and active protection of Māori and iwi-led health providers |
|  | Acknowledging iwi/ Māori (Tangata Whenua) as te Tiriti partner | Recognition of te Tiriti responsibilities for the health system | Acknowledgement and awareness of alliance’s obligation to respond to te Tiriti principles | Key actions are aligned with five principles of te Tiriti: self-determination, equity, active protection, partnership, and options for Māori models of care | Alliance is continually monitoring, reviewing, and reporting on how well they are responding to actions based on five te Tiriti principles |
| Areas to work on: | | | | | |

| Key element 3 - Clinical leadership and involvement  A system through which health care professionals provide leadership and system oversight with a focus on continuous quality improvement to create an environment for evidence-based clinical practice and team-based approaches to care delivery^[[2]](#footnote-2)^. | | | | | |
| --- | --- | --- | --- | --- | --- |
| Outcome descriptor (what does the established element demonstrate?) | **Indicators (What will show this?)** | **Maturity scale – what we expect to see at each stage** | | | |
|  |  | **Beginning** | **Emerging** | **Established (this is what good looks like)** | **Excellence (outstanding eg health and social integration)** |
| Transformational efforts are designed and implemented with or are led by the clinical leaders and health care professionals. Recommended solutions are accepted and implemented by the operational leaders | Operational and clinical leadership work as one | Rebalancing of managerial and clinical input into system change development | Trust model in place between managerial and clinical input and understanding of the different roles | Clinicians involved in investment decisions  High attendance of clinical leaders to alliance meetings  Explicit sign off by clinicians | Distributed leadership and shared vision through honest conversations, respect, acknowledgement of roles, and sustainable ongoing leadership |
|  | The degree to which clinicians’ and managers’ knowledge of population health data is understood and actioned | Little or no evidence of population health data being considered by managers and clinicians | Some evidence of meaningful discussion between clinicians and managers about equity issues | Work programme has alignment between population health data and funding allocation | Clear evidence of regular consideration of equity issues between clinicians and managers and clearly documented improved health outcomes |
|  | Membership and way of working reflects te Tiriti principles | Little or no Māori clinical leadership | Māori clinicians and managers part of clinical and alliance forums | Evidence of Māori clinical attendance, participation, and influence in investment decisions | Dedicated workstream for Māori outcomes with multi-disciplinary and -sectorial approach |
| Areas to work on: | | | | | |

| Key element 4 – Involved people, whanau, and community  An approach that actively involves individuals, carers, hapū, whānau, iwi and communities in the design and delivery of health care to generate significant benefits to the health care and wellbeing of all people. | | | | | |
| --- | --- | --- | --- | --- | --- |
| Outcome descriptor (what does the established element demonstrate?) | **Indicators (What will show this?)** | **Maturity scale – what we expect to see at each stage** | | | |
|  |  | **Beginning** | **Emerging** | **Established (this is what good looks like)** | **Excellence (outstanding eg health and social integration)** |
| People, whānau and communities are involved at the governance and decision-making levels to co-design the system and the services | Information is available to communities to understand what is important to them | Acknowledge the need to have the right people around the table | Acknowledgement that engagement is broader than a seat at the table, i.e., it is how the alliance engages with its broader community to prioritise and exchange information | A multi-channel engagement plan in place with meaningful input from individuals and communities that leads them to believe that the right decisions are being made for services they need | Individuals and communities are involved in decision-making for re-prioritising services to changing need |
|  | Degree to which individual, whānau and community voice from all populations is heard and acted upon in decision-making | Priority populations are informed of decisions through information provided by the alliance | Alliance consults priority population communities on draft proposals but makes the final decisions | Community representatives from priority populations part of alliance and influencing decision-making of the alliance | Individuals and communities from priority populations are involved in co-design and decision-making processes of the alliance |
|  | Tangata Whenua (Māori, iwi, whānau, hapū) are partners in all stages of design, delivery, and implementation | Tangata Whenua are informed of decisions through information provided by the alliance | Alliance consults Tangata Whenua on draft proposals but makes the final decisions | Alliances collaborates with Tangata Whenua to develop solutions together and Tangata Whenua are involved in decision-making process or make joint decisions with the alliance | Alliance partners with Tangata Whenua to co-design services and implements decisions made by Tangata Whenua for the final solution |
| Areas to work on: | | | | | |

| Key element 5 – Integrated health information  The availability of technology, and health and social information, both at identifiable and population aggregate level, across the different parts of the system at local and national level. This readily accessible health information is responsive to needs and guides commissioning decisions. | | | | | |
| --- | --- | --- | --- | --- | --- |
| Outcome descriptor (what does the established element demonstrate?) | **Indicators (What will show this?)** | **Maturity scale – what we expect to see at each stage** | | | |
|  |  | **Beginning** | **Emerging** | **Established (this is what good looks like)** | **Excellence (outstanding e.g., health and social integration)** |
| DHBs can link data from multiple platforms that includes administrative and social sector data at the population and patient identifiable level and share with the alliance for continuous improvement | NHI linked data available to meet decision-making needs for the problem at hand | Alliance is receiving basic data from the DHB | DHB is supporting alliance with data sharing agreements with all system partners | Alliance has access to real-time data from across the system, both at NHI and population level | Alliance has access to the right information at the right time for forecasting and feedback to drive continuous improvement |
|  | Degree to which integrated information and intelligence for high priority populations are explicitly considered and managed routinely | Alliance receives basic data at DHB level disaggregated by age, gender, and ethnicity | Alliance receives ad hoc integrated data at national and local level, disaggregated by age, gender and ethnicity | Alliance routinely receives timely, useful, integrated data as part of mandatory reporting and monitoring by ethnicity | Alliance has access to health information that is integrated with administrative and social sector data to drive equity actions |
|  | Māori have sovereignty over integrated health information | Alliance receives basic data at DHB level disaggregated by age, gender, and ethnicity | Alliance receives ad hoc integrated data at national and local level, disaggregated by age, gender and ethnicity with recognition of Māori data sovereignty | Alliance routinely receives timely, useful, integrated data as part of mandatory reporting and monitoring by ethnicity | Alliance has access to health information that is integrated with administrative and social sector data to meet their te Tiriti obligations |
| Areas to work on: | | | | | |

| Key element 6 – Analytic capability  The availability of technology and the ability to access real-time and trend information, analyse, link clinical and administrative data, and produce insights and evidence for frontline staff to measure, understand and feedback data on clinical variation and outcomes. | | | | | |
| --- | --- | --- | --- | --- | --- |
| Outcome descriptor (what does the established element demonstrate?) | **Indicators (What will show this?)** | **Maturity scale – what we expect to see at each stage** | | | |
|  |  | **Beginning** | **Emerging** | **Established (this is what good looks like)** | **Excellence (outstanding e.g., health and social integration)** |
| Improvement culture, skills, and knowledge available to turn all intelligence about health of local populations into insights about variation and change in quality and outcomes of health care for alliance to use for continuous improvement | Intelligence about health of local populations into insights about variation and change in quality and outcomes of health care | Recognition that intelligence is required and initial discussion to establish analytic needs for alliance work programme | DHB invests in tools and resources to produce evidence for alliance to use to drive decisions | Growing sophistication on the level of insights and evidence available to enable alliance to conduct consistent analysis over time to plan, do, study and act | Alliance is able to use insights and evidence for ‘rear-view’ and for forward projection  Cross district learning and sharing is occurring |
|  | Degree to which intelligence resources are allocated to analysing data with an equity focus | Limited analytic capacity and little understanding of analysing variation or measuring change for high priority populations | Appropriate analytic capacity and capability to support alliance with intelligence to analyse variation and measure change for high priority populations | Insights and evidence are consistently provided to alliance to routinely plan, do, study and act for high priority populations | Insights and evidence are driving investment decisions of the alliance to respond to the future needs of high priority populations |
|  | Degree to which intelligence resources are allocated to meet te Tiriti obligations | Limited analytic capacity to understand, measure and routinely report Māori Health outcomes | Appropriate analytic capacity and capability to support alliance with intelligence to understand, measure and routinely report Māori Health outcomes | Insights and evidence are consistently provided to alliance to understand, measure, and routinely report Māori Health outcomes | Alliance becomes intuitive to the future needs of Māori for the next 10-15 years |
| Areas to work on: | | | | | |

| Key element 7 – Dedicated resources and time  Availability of appropriate continuous resourcing such as appropriate health workforce, funding, knowledge, time, project management support, and administration support. There is an acknowledgement that transformational change needs capacity and long-term commitment. | | | | | |
| --- | --- | --- | --- | --- | --- |
| Outcome descriptor (what does the established element demonstrate?) | **Indicators (What will show this?)** | **Maturity scale – what we expect to see at each stage** | | | |
|  |  | **Beginning** | **Emerging** | **Established (this is what good looks like)** | **Excellence (outstanding e.g., health and social integration)** |
| Sustainable and dedicated resources located in the right place in the system for change management | Resources ring-fenced to manage and support change management and decision-making | Alliancing is seen as a good idea but embryonic form, function, and dedicated resources  relies on goodwill | DHB recognises that alliancing needs to be resourced within the system with funding and human resources | Alliance has an independent programme office with dedicated management and clinical resource to achieve its work programme | Alliance programme office is embedded in the system with sustainable funding |
|  | Degree to which equity is explicitly incorporated into all elements of alliance work programme | Equity mentioned in alliance terms of reference but no further evidence of funding | Mention of equity in alliance related work with some funded initiatives aimed at improving equity | Multiple funded equity actions aimed at high priority populations with routine monitoring and reporting of health outcomes | Equity is at the centre of all the alliance work programme with sustained funding, and routine monitoring and reporting of health outcomes |
|  | Core business spending is challenged and re-prioritised to meet Māori aspirations for wellbeing | Alliance recognises the need to prioritise resources for equity actions to improve Māori health outcomes | Alliance has funded equity actions to improve Māori health outcomes | Alliance’s decisions on funding and resource allocations are equity based within the context of te Tiriti | Alliance challenges current investment decisions to support hauora Māori models of care |
| Areas to work on: | | | | | |

| Key element 8 – Intelligent commissioning  Commissioning is the process of continuously developing services and committing resources to enable the best health outcomes and wellbeing and that includes many activities ranging from health needs assessment, cultural paradigms, and development of pathways to service specification and contract management or procurement, underpinned by continuous improvement. | | | | | |
| --- | --- | --- | --- | --- | --- |
| Outcome descriptor (what does the established element demonstrate?) | **Indicators (What will show this?)** | **Maturity scale – what we expect to see at each stage** | | | |
|  |  | **Beginning** | **Emerging** | **Established (this is what good looks like)** | **Excellence (outstanding e.g., health and social integration)** |
| Continuous strategic and operational planning is underpinned by continuous improvement that enables the alliance to make evidence-based decisions | Clear evidence for alliance decision-making for commissioning of health services | Alliance recognise the need for evidence-based decision-making for commissioning of health services | Alliance has access to limited evidence to inform their decision-making for current and future commissioning of health services | Alliance has access to comprehensive evidence to inform some of their decision-making for current and future commissioning of health services | Alliance is using the evidence to inform all their decision-making for current and future commissioning of health services |
|  | Prioritisation of resources in proportion to high priority populations’ need and risk with a targeted approach to equity | Alliance decisions are not based on available intelligence and there is no consideration of priority populations (perpetuating status quo) | Some decisions are based on evidence to prioritise resources in proportion to high priority populations’ need and risk | Equity is at the centre of all decision-making for current and future commissioning of health services | Continuous use of evidence, monitoring of actions, and reporting to show that current and future commissioning is proportional to high priority populations’ need and risk |
|  | Prioritisation of resources in proportion to Māori need and risk with a tailored and target approach to actively protecting Māori wellbeing | Alliance acknowledging that resources to Māori will be different | Alliance is identifying resources in proportion to Māori need and risk to improve Māori wellbeing | Alliance is recommending to DHB to de-invest in other areas and re-invest into Māori and prioritise new funding to Māori | Alliance is recommending commissioning decisions based on kaupapa Māori models and protecting Māori and iwi-led health providers |
| Areas to work on: | | | | | |

| Key element 9 – Understanding of equity  Equity recognises different people with different levels of advantage require different approaches and resources to get equitable health outcomes^[[3]](#footnote-3)^. Emphasis is given to Pasifika and other high opportunity populations such those with mental health conditions and with disability. | | | | | |
| --- | --- | --- | --- | --- | --- |
| Outcome descriptor (what does the established element demonstrate?) | **Indicators (What will show this?)** | **Maturity scale – what we expect to see at each stage** | | | |
|  |  | **Beginning** | **Emerging** | **Established (this is what good looks like)** | **Excellence (outstanding e.g., health and social integration)** |
| Alliance is able to identify and understand the variation in health care in their population, identify population groups that consistently experience poor health outcomes and re-prioristise activities and funding to address health inequities | Every alliance decision has an equity focus based on information that is easy to understand | Equity mentioned but not yet widely understood or explicitly addressed | Start to see equity as key part of regular alliance conversation including discussion on how to address inequities | Alliance work programme and investment decisions explicitly address health inequities and undertake routine monitoring of effectiveness of change to close gaps | Alliance works collaboratively with social sector partners to reduce inequities including a focus on social determinants of health |
|  | Degree to which alliance uses proportionate universalism approach | Alliance understands that actions and resources need to be proportionate to need and level of disadvantage in their population | Alliance identifies high priority populations in their district | Alliance prioritises their work programme and resources proportionate to the need of their high priority populations | Evidence of re-prioritisation of activities and resources Pasifika and other population groups that experience health inequities, which leads to documented improved health outcomes |
|  | Degree to which te Tiriti is understood in the modern-day health system (ie biculturalism in a multi-culturalism context) | Understanding of root causes of inequities within the context of te Tiriti | Alliance is able to conduct basic analysis to understand inequities for Māori (i.e. by age and gender) | Alliance is using inter-sectional analysis to drive key actions to address inequities within the context of te Tiriti | Evidence of re-prioritisation of activities and resources for Māori within the context of te Tiriti, which leads to documented improved health outcomes for Māori and active protection of Māori and iwi-led health providers |
| Areas to work on: | | | | | |

| Key element 10 – Continuous improvement  Systematic and sustained use of continuous quality improvement methods, measurement tools and feedback loops that provide opportunities for learning and build accountability in the system. | | | | | |
| --- | --- | --- | --- | --- | --- |
| Outcome descriptor (what does the established element demonstrate?) | **Indicators (What will show this?)** | **Maturity scale – what we expect to see at each stage** | | | |
|  |  | **Beginning** | **Emerging** | **Established (this is what good looks like)** | **Excellence (outstanding e.g., health and social integration)** |
| Improvement culture, skills, knowledge, and experience of using quality improvement science and techniques to drive measurable impact in quality and outcomes of the health system by the alliance | Specific resource and funding put aside for alliances to use for continuous improvement | Recognition that conscious investment in continuous improvement is essential | Alliance has resources for continuous improvement | Continuous improvement activities are part of every decision and implementation with continued feedback loops | Continuous improvement builds accountability in the system to measure progress towards achieving the Triple Aim |
|  | Degree to which alliance can demonstrate equity gap is reducing as a result of improvement activities (no minimum targets) | Improvement activities have had no effect on inequities or worsened | Existing or new improvement activities consider inequity, but effects are unclear or inconsistent | Existing and new improvement activities consistently focused on improvements in equity | Accountability in the system through routine measuring, monitoring, and reporting of activities to reduce inequities for high priority populations |
|  | Māori aspirations are integrated in improvement journey | Awareness that traditional improvement methods and models are not always appropriate for Māori | Identification of Māori improvement models e.g., Te whare tapa whā model | Māori knowledge is respected, and Māori measures of wellbeing are defined by Tangata Whenua | Alliance is empowering Māori to define their own priorities for wellbeing and realise their aspirations |
| Areas to work on: | | | | | |

**Alliance Action Plan (post self-assessment)**

| Key element | Improvement focus area | Actions | Responsibility | Timeline |
| --- | --- | --- | --- | --- |
|  |  |  |  |  |
|  |  |  |  |  |
|  |  |  |  |  |
|  |  |  |  |  |
|  |  |  |  |  |
|  |  |  |  |  |
|  |  |  |  |  |
|  |  |  |  |  |
|  |  |  |  |  |
|  |  |  |  |  |

1. Waitangi Tribunal. Report on Stage One of the Health Services and Outcomes Kaupapa Inquiry. New Zealand: Waitangi Tribunal WAI 2575, 2019. <https://www.waitangitribunal.govt.nz/inquiries/kaupapa-inquiries/health-services-and-outcomes-inquiry/> [↑](#footnote-ref-1)
2. Gauld R, Horsburgh S, Flynn M, et al. Do different approaches to clinical governance development and implementation make a difference? Findings from Ireland and New Zealand. Journal of Health, Organisation and Management 2017;**31**(7/8):682-95 [↑](#footnote-ref-2)
3. Ministry of Health. Achieving equity. 2019. https://[www.health.govt.nz/about-ministry/what-we-do/work-programme-2018/achieving-equity](http://www.health.govt.nz/about-ministry/what-we-do/work-programme-2018/achieving-equity) [↑](#footnote-ref-3)
